# A biomimetic approach to modulating the sustained release of fibroblast growth factor 2 from fibrin microthread scaffolds

**DOI:** 10.37349/ebmx.2024.00006

**Published:** 2024-04-23

**Authors:** Meagan E. Carnes, Cailin R. Gonyea, Jeannine M. Coburn, George D. Pins

**Affiliations:** Department of Biomedical Engineering, Worcester Polytechnic Institute, Worcester, MA 01609, USA

**Keywords:** Fibroblast growth factor 2, fibrin microthreads, fibrin, tissue engineering, skeletal muscle, myoblast

## Abstract

**Aim:**

The pleiotropic effect of fibroblast growth factor 2 (FGF2) on promoting myogenesis, angiogenesis, and innervation makes it an ideal growth factor for treating volumetric muscle loss (VML) injuries. While an initial delivery of FGF2 has demonstrated enhanced regenerative potential, the sustained delivery of FGF2 from scaffolds with robust structural properties as well as biophysical and biochemical signaling cues has yet to be explored for treating VML. The goal of this study is to develop an instructive fibrin microthread scaffold with intrinsic topographic alignment cues as well as regenerative signaling cues and a physiologically relevant, sustained release of FGF2 to direct myogenesis and ultimately enhance functional muscle regeneration.

**Methods:**

Heparin was passively adsorbed or carbodiimide-conjugated to microthreads, creating a biomimetic binding strategy, mimicking FGF2 sequestration in the extracellular matrix (ECM). It was also evaluated whether FGF2 incorporated into fibrin microthreads would yield sustained release. It was hypothesized that heparin-conjugated and co-incorporated (co-inc) fibrin microthreads would facilitate sustained release of FGF2 from the scaffold and enhance *in vitro* myoblast proliferation and outgrowth.

**Results:**

Toluidine blue staining and Fourier transform infrared spectroscopy confirmed that carbodiimide-conjugated heparin bound to fibrin microthreads in a dose-dependent manner. Release kinetics revealed that heparin-conjugated fibrin microthreads exhibited sustained release of FGF2 over a period of one week. An *in vitro* assay demonstrated that FGF2 released from microthreads remained bioactive, stimulating myoblast proliferation over four days. Finally, a cellular outgrowth assay suggests that FGF2 promotes increased outgrowth onto microthreads.

**Conclusions:**

It was anticipated that the combined effects of fibrin microthread structural properties, topographic alignment cues, and FGF2 release profiles will facilitate the fabrication of a biomimetic scaffold that enhances the regeneration of functional muscle tissue for the treatment of VML injuries.

## Introduction

A total of 65.8 million Americans suffer from musculoskeletal injuries annually, costing approximately 176 billion dollars to treat which profoundly impacts the quality of life of patients [1–5]. These traumatic injuries known as volumetric muscle loss (VML) are characterized by a large-scale injury that results in limited functional recovery. Skeletal muscle is capable of endogenous regeneration through activation of resident progenitor cells known as satellite cells, but this ability is greatly reduced in VML. These large-scale injuries destroy native signaling cues and growth factor reservoirs such as the basement membrane and connective tissue, hindering the damaged tissue’s ability to direct regeneration. Instead of regenerating functional, contractile muscle, non-functional scar tissue is deposited to fill the void. The current standard of care for VML injuries is autologous tissue transfer, where a muscle flap is excised from an undamaged muscle and grafted into the injury [6]. This procedure is complicated, time-consuming, and has exhibited limited ability to restore function [7, 8]. Additionally, approximately 10% of muscle flap procedures result in complications such as infection or donor site morbidity due to tissue necrosis [6]. As such, there is a need for an alternative treatment for VML capable of enhancing functional muscle regeneration.

Growth factors serve to guide regeneration *in vivo* and have been shown to promote functional muscle regeneration when delivered to an injury, thus representing a promising therapeutic strategy for treating VML [9–19]. Fibroblast growth factor 2 (FGF2) is an ideal growth factor for treating skeletal muscle injuries due to its pleiotropic effects on myogenesis, angiogenesis, and innervation [20]. After injury, the basement membrane releases heparan sulfate proteoglycan-bound growth factors such as FGF2 [21–25]. FGF2 is responsible for stimulating the proliferation and migration of activated satellite cells [26–29], and is present in injured muscle tissue 2–8 days after injury and peaks at 6–8 days [30]. FGF2 has also been shown to stimulate endothelial migration and sprouting, as well as pericyte and smooth muscle cell migration [31]. *In vivo*, FGF2 stimulates the formation of more mature vessels than other proangiogenic growth factors such as vascular endothelial growth factor (VEGF) [32]. Additionally, FGF2 has been shown to have neurotrophic activity, stimulating the synthesis and secretion of nerve growth factor (NGF), and promoting neuronal survival and outgrowth [20, 33–36]. Taken together, these findings suggest that FGF2 is a promising growth factor for treating complex, multi-tissue VML injuries.

Initially, FGF2 was investigated for the clinical treatment of cardiovascular disease, with the goal of stimulating revascularization in ischemic heart regions [37]. However, early phase II clinical trials delivering FGF2 via bolus intra-coronary or intra-arterial infusion did not demonstrate therapeutic effectiveness at trial end points [38, 39]. This was a result of an inability to sustain physiologically relevant levels of FGF2 for the necessary time frame, as well as its short *in vivo* half-life on the order of minutes [37, 40]. To mitigate these issues, biomaterial-based strategies have been employed that either chemically immobilize or physically encapsulate growth factors within a polymer matrix, sustaining their release over a clinically-relevant time frame and preventing their denaturation [41]. One method of chemical immobilization uses heparin, a glycosaminoglycan that binds with high affinity to certain growth factors including FGF2 [21, 22, 42, 43]. Heparin sulfate is a proteoglycan found in the basement membrane that acts as a reservoir for growth factors essential for myogenesis, including FGF2 [21, 23, 44]. Heparan sulfate also significantly enhances FGF2 signaling, binding to both the growth factor and its receptor, forming a ternary complex [45, 46]. Heparin-conjugated scaffolds provide controlled release of growth factors and mimic the presentation of growth factors in the extracellular matrix (ECM) [21, 22, 32, 35, 43, 47]. A phase I clinical trial delivered sustained release of FGF2 via heparin-alginate microcapsules implanted in ischemic myocardial regions of patients [48]. Significant improvement in perfusion after 90 days was demonstrated in the high-dose FGF2 group compared with controls. A three-year follow-up study found patients treated with heparin-delivered FGF2 had significantly lower symptom recurrence, indicating a prolonged revascularization due to sustained heparin-mediated FGF2 release [49].

In a more recent clinical study, the therapeutic effects of sustained FGF2 release have been applied in early clinical trials to treat ischemic skeletal muscle tissue [50–52]. Two-phase I-IIa studies investigating the safety and efficacy of FGF2-incorporated gelatin hydrogel microspheres delivered via intramuscular (IM) injection to patients with critical limb ischemia (CLI) demonstrated significant improvements in primary endpoints compared to pretreatment [50, 52]. Another pilot clinical study tested the safety and efficacy of IM injection of FGF2-incorporated hydrogels on patients with CLI, and found no adverse events and improved ischemic symptoms compared to pretreatment at the trial end point [51]. While the sustained release of FGF2 shows promise in treating ischemic skeletal muscle injuries, its use in treating the more complex, multi-tissue injuries incurred in VML is still actively under clinical investigation.

Several groups have investigated delivering FGF2 to VML defects in preclinical animal models. Multiple biomaterial scaffold strategies to deliver FGF2 to these injuries have been investigated, including delivering FGF2 alone [11, 12, 17, 53], FGF2 in tandem with cells [11, 12, 53–55], FGF2 transgenes [56], and myoblasts overexpressing FGF2 [57]. When plasmid and adenoviral vectors encoding FGF2 immobilized in a collagen-gelatin hydrogel were delivered to rat quadriceps injuries, *FGF2* gene delivery enhanced the number of clusters of differentiation 31 positive (CD31^+^) endothelial cells and CD56^+^ myotubes compared to matrix alone, indicating enhanced angiogenesis and myogenesis in FGF2-treated wounds [56]. Another study evaluated the delivery of FGF2-overexpressing myoblasts encapsulated within alginate spheres to a soleus muscle crush injury in a rat [57]. They found that delivery of FGF2 overexpressing myoblasts increased microvessel density and skeletal muscle cell proliferation at 4 days after injury. Other studies have utilized FGF2 delivery to enhance transplanted myoblast or stem cell survival and proliferation [53, 55]. Co-delivery of FGF2 and human adipose-derived stem cells resulted in functional recovery, revascularization, reinnervation, and minimal fibrosis in a murine VML laceration injury [53]. Co-delivery of FGF2 and myoblasts in gelatin microspheres to a rat muscle injury showed significantly enhanced relative gene expression levels of CD31 and myogenin in FGF2/myoblast scaffolds compared to myoblasts alone or unloaded gelatin microspheres [55]. Similarly, polymeric delivery of FGF2 alone to rat VML defects has demonstrated enhanced host cell infiltration and myofiber regeneration [17], as well as functional recovery [12]. Taken together, FGF2 has shown marked enhancement of myogenesis, revascularization, and reinnervation when delivered alone or in tandem with cells to a VML injury. While this work demonstrates the therapeutic potential of sustained FGF2 delivery, it has primarily utilized hydrogel scaffolds, which lack the essential mechanical support and aligned contact guidance cues of fiber-based scaffolds [9, 58, 59]. The delivery of FGF2 from polymer scaffolds with robust mechanical properties and topographic alignment cues has yet to be explored for treating VML injuries. Towards this goal, the investigator’s laboratory developed a series of bioprinting and crosslinking strategies to fabricate fibrin microthreads with precisely tuned mechanical properties that promote functional skeletal muscle regeneration in small animal defect models of VML injuries [60–67]. However, there remains an unmet need to investigate the controlled release of FGF2 from these microthread scaffolds to enhance cell-mediated myogenesis.

The goal of this study is to develop an instructive fibrin microthread scaffold with intrinsic topographic alignment cues that provide a physiologically-relevant sustained release of FGF2. To accomplish this, the investigators passively adsorbed or covalently conjugated through carbodiimide-mediated coupling heparin to the surface of fibrin microthreads, creating a biomimetic binding strategy recapitulating FGF2 sequestration in the basement membrane. Fibrin is an ideal scaffold material for incorporating FGF2, as it binds with high affinity to FGF2 and protects it from proteolytic degradation [68]. As such, the investigators also assessed whether co-incorporating FGF2 into fibrin microthread scaffolds by mixing prior to extrusion would promote sustained release of FGF2. The investigators evaluated the effect of both FGF2 incorporation techniques on release kinetics as well as myoblast proliferation and outgrowth. The investigators hypothesized that heparin-conjugated and co-incorporated (co-inc) fibrin microthreads would facilitate a sustained physiologic release of FGF2 from the scaffold and enhance *in vitro* myoblast proliferation and outgrowth.

## Materials and methods

### Fibrin microthread extrusion

Fibrin microthreads were formed by co-extruding fibrinogen and thrombin solutions as described previously [69]. Briefly, fibrinogen isolated from bovine plasma (MP Biomedicals, CAT: 8820224) was dissolved in *N*-2-hydroxyethyl-piperazine-*N*’−2-ethanesulfonic acid (HEPES, Sigma-Aldrich, CAT: H3375) buffered saline [HBS, 20 mmol/L HEPES, 0.9% NaCl (Sigma-Aldrich, CAT: S9888), pH 7.4] to a concentration of 70 mg/mL and stored at −20°C until use. Thrombin isolated from bovine plasma (Sigma Aldrich, CAT: T7009) was dissolved in HBS at 40 U/mL and stored at −20°C until use. To create microthreads, fibrinogen and thrombin solutions were thawed to room temperature (RT), and thrombin was mixed with a 40 mmol/L calcium chloride (CaCl_2_) solution (Sigma-Aldrich, CAT: C5670) to form a 6 U/mL working solution. Equal volumes of fibrinogen and thrombin solutions were taken up separately into 1 mL syringes and inserted into the tip of a blending applicator (Micromedics Inc., SA-3670). The solutions were mixed in the blending applicator and extruded through polyethylene tubing with an inner diameter of 0.86 mm into a 10 mmol/L HEPES buffer bath (pH 7.4) in a Teflon-coated pan. Threads were extruded at 0.225 mL/min using a dual syringe pump. After microthreads were incubated for 10–15 min to allow for polymerization, the scaffolds were removed and stretched to approximately 300% of their initial drawn length and hung overnight to dry under the tension of their own weight.

Fibrin microthreads co-inc with FGF2 (co-inc) were created as previously described [70]. Recombinant human FGF2 (Peprotech Inc., CAT: BFF-H4117) stock solution was mixed with 70 mg/mL fibrinogen precursor solution to reach a final FGF2 concentration of 1 μg/mL prior to co-extrusion with thrombin. Microthread extrusion, polymerization, and stretching followed the same procedure as described above for uncross linked microthreads (UNX).

### Fibrin film generation

Fibrin films were used to evaluate heparin binding due to their ease of production and analysis. Fibrin films are generated by mixing 1:1 (v:v) 70 mg/mL fibrinogen with 6 U/mL thrombin, the same components coextruded 1:1 through a blending applicator to make fibrin microthreads. A non-adhesive casting surface was created by coating a polydimethylsiloxane (PDMS, Dow Sylgard 184, Elsworth Adhesives) sheet with 1% Pluronic-F127 (Sigma Aldrich, CAT: P2443) in deionized water (dH_2_O) for 30 min. Immediately after mixing fibrinogen and thrombin solutions together, 150 μL was cast on top of square vellum frames (1.2 cm × 1.2 cm), which were placed on top of Pluronic-coated PDMS. After 30 min of polymerization gels were carefully peeled off PDMS, rinsed three times for 5 min in dH_2_O to de-salt, and dried overnight to form a film.

### Heparin conjugation to fibrin microthreads and films

To create a biomimetic conjugation strategy to tether FGF2 to fibrin microthreads, heparin was immobilized to fibrin microthread surfaces with or without carbodiimide crosslinking. UNX fibrin microthreads were suspended in 1-well dishes by inserting them between two slatted PDMS bars located on the edges of the dish. Approximately thirteen distinct 5 cm long microthreads were suspended in each 1-well dish. Microthreads to be crosslinked were hydrated in 30 mL of 100 mmol/L sodium phosphate buffer (NaH_2_PO_4_, pH 7.4, Sigma Aldrich, CAT: S0751) for 30 min at RT. After 30 min, hydrating buffers were removed and replaced with 100 mmol/L NaH_2_PO_4_ containing 28 mmol/L 1-ethyl-3-(3-dimethyl aminopropyl) carbodiimide (EDC, Sigma Aldrich, CAT: E6383), 16 mmol/L *N*-hydroxysuccinimide (NHS, Sigma Aldrich; CAT: 130672), and heparin sodium salt porcine submucosa (HEP, Calbiochem, CAT: 375095) at a final concentration of 0 μg/mL (EDC), 10 μg/mL (EDC-HEP 10), 100 μg/mL (EDC-HEP 100), or 1,000 μg/mL (EDC-HEP 1000). After 2 h in crosslinking solution, the EDC/NHS solution was aspirated and microthreads were rinsed three times for 5 min with dH_2_O. Next, a 0.5% glycine in phosphate-buffered saline (PBS; Thermo-Fisher, CAT: 10010023) solution was added to microthreads for 1 h to extract the NHS reaction product and quench any residual activated carboxylic acid groups [71, 72]. After 1 h, the glycine solution was removed and the threads were rinsed twice for 5 min with dH_2_O. Suspended microthreads were allowed to dry overnight in tension. Previous studies showed that EDC crosslinking does not significantly decrease cell viability [66].

Control microthreads that remained uncross linked were hydrated with PBS for 30 min at RT. After 30 min, PBS was removed and replaced with 30 mL of 10 μg/mL (UNX-HEP 10), or 1,000 μg/mL (UNX-HEP 1000) HEP in PBS. After 2 h, the heparin solution was aspirated and microthreads were rinsed three times for 5 min with dH_2_O. Next, a 0.5% glycine in PBS solution was added to microthreads for 1 h, then removed and two rinses for 5 min with dH_2_O were performed. Suspended microthreads dried overnight under the tension of their own weight.

A final condition evaluated fibrin microthreads that were first EDC crosslinked and subsequently adsorbed with HEP (EDC then HEP 1000). Microthreads were hydrated in 30 mL of 100 mmol/L NaH_2_PO_4_ (pH 7.4) for 30 min at RT. After 30 min, hydrating buffers were removed and replaced with 100 mmol/L NaH_2_PO_4_ containing 28 mmol/L EDC and 16 mmol/L NHS for 2 h. Next, the EDC/NHS solution was aspirated and microthreads were rinsed three times for 5 min with dH_2_O. After rinses, a 0.5% glycine in PBS solution was added to microthreads for 1 h, then removed and two rinses for 5 min with dH_2_O were performed. Next, 30 mL of 1,000 μg/mL HEP in PBS was added to microthreads for 2 h, followed by two rinses for 5 min with dH_2_O. Suspended microthreads dried overnight under the tension of their own weight. Fibrin films underwent the same heparin conjugation method with or without crosslinking described above for the fibrin microthreads.

### Toluidine blue staining of heparin-conjugated scaffolds

Toluidine blue staining was performed to assess the degree of heparin conjugation to fibrin films. Toluidine blue binds with high affinity to heparin via electrostatic interactions and is a commonly employed method of assessing heparin incorporation in scaffolds [43, 73–75]. To perform staining, heparin conjugated fibrin films were placed in toluidine blue stain [0.1 mol/L HCl (Sigma-Aldrich, CAT: 320331), 2 mg/mL NaCl (Sigma-Aldrich, CAT: S9888), 0.4 mg/mL toluidine blue (Sigma Aldrich, CAT: 89640)] for 4 h at RT, as previously described [74]. After 4 h, films were then rinsed two times with dH_2_O and allowed to dry. Dried films were placed on a white backdrop and imaged on a Nikon SMZ-U stereo microscope (Nikon, Model SMZ-U). To quantify changes in dye uptake between microthread conditions, the pixel intensity of the images was quantified using ImageJ (release version 1.52t) by converting images to 8-bit grayscale and determining the mean grey value from three (3)-144 × 144 square pixel regions on the film. Mean grey values of the image background from three (3)-144 × 144 square pixel regions were subtracted to normalize data. All conditions were run in duplicate for each experimental replicate, and a total of three experimental replicates were performed.

### Fourier transform infrared spectroscopy

Fourier transform infrared spectroscopy (FTIR) was used as an additional analytical tool to characterize heparin conjugation to fibrin film scaffolds by confirming that the carboxyl groups of heparin reacted with the amine groups of fibrin [43, 73, 76, 77]. Dried fibrin film samples were positioned onto the attenuated total reflectance (ATR) crystal of a Bruker Vertex 70 instrument (Bruker, Vertex Model 70) with a Golden Gate ATR accessory (Specac, Golden Gate ATR). FTIR absorbance spectra data were collected in the mid-infrared (mid-IR) range, 4,500−800 cm^−1^, and obtained by averaging 1,024 scans. Backgrounds were subtracted from each spectrum. Three batches of films for each condition were analyzed and averaged to obtain a representative spectrum for comparison to other conditions. Baseline correction of absorbance was performed by normalizing data at 4,200 cm^−1^, a region of the spectra with no characteristic peaks. To assess whether an amide link was formed through heparin conjugation, amide I, amide II, and amide III bands were evaluated [43, 73, 76, 77]. Amide I (approximate 1,630 cm^−1^) peaks are primarily a result of C=O stretching, while the amide II (approximate 1,520 cm^−1^) peak is a result of N-H bending and C-N stretching [73, 76, 77]. Sulfonated groups and amide III are characterized by a peak at 1,240 cm^−1^ [43, 73, 76, 77].

### Determine FGF2 release from fibrin microthreads

To quantify FGF2 release from fibrin microthreads over time, an FGF2-specific enzyme-linked immunosorbent assay (ELISA, Peprotech Inc., CAT: 900-K08) was performed. Microthread samples were prepared by anchoring ten (10)-1.8 cm-long microthreads of the same condition side-by-side onto a medical-grade stainless steel ring (1.8 cm inner diameter) with medical-grade silicone adhesive. Each ring was placed in a separate well of a 6-well dish, where it was blocked in 0.25% bovine serum albumin (BSA) in PBS for 1 h, then replaced with 1 mL of a 3.5 μg/mL solution of human FGF2 (Peprotech Inc., CAT: 100-18B) in sterile PBS. Microthread rings were incubated in FGF2 solution for 16 h at RT, then the solution was aspirated from wells and excess FGF2 was carefully blotted off stainless-steel rings. Immediately after removing excess FGF2, 1 mL of sterile PBS was added to samples, each remaining in its own well of a 6-well dish. Plates were sealed with parafilm and placed in a 37°C oven on a shaker plate for continuous agitation. This denoted the start time for the release assay. To evaluate the release of FGF2 over time, PBS samples were collected at 1h, 4 h, 8 h, 1 day, 2 days, 3 days, 4 days, 5 days, 6 days, and 7 days after initiation of the release study, and stored at −20°C in low-bind microcentrifuge tubes.

After all samples were collected, an FGF2-specific ELISA was performed in accordance with the manufacturer’s instructions. Briefly, ELISA plates were coated with capture antibody overnight, blocked in blocking buffer for 1 h, and then incubated with appropriately diluted samples for 2 h. After the specific binding of sample antigens, a detection antibody was added to plates for 2 h, followed by enzyme-linked avidin-horseradish peroxidase (HRP) which reacted for 30 min. Finally, 2,2’-azino-bis-3-ethylbenzothiazoline-6-sulfonate (ABTS) substrate was added to produce a soluble, colored product. Plates were read at 405 nm and 650 nm on a spectrophotometer (SpectraMax 250, Molecular Devices) so a wavelength subtraction of 405–650 nm could be performed before converting optical density readings to FGF2 concentration. Finally, the cumulative release of two control samples was subtracted from other samples; these controls were UNX microthreads that did not receive FGF2 and a stainless-steel ring with silicone glue and no microthreads that were passively adsorbed with FGF2.

### Cell culture

C2C12 immortalized mouse myoblasts (ATCC, CRL-1772) were cultured in complete medium (CM), consisting of a 1:1 (v/v) ratio of high glucose Dulbecco’s modified Eagle medium (DMEM, Gibco, CAT: 10313–021) and Ham’s F12 (Gibco, CAT: 11765054), supplemented with 4 mmol/L *L*-glutamine (Gibco, CAT: 35050–061), 10% fetal bovine serum (FBS, HyClone, CAT: SH30071.03), 100 U/mL penicillin (Thermo Fisher Scientific, CAT: 15140122), 100 μg/mL streptomycin (Thermo Fisher Scientific, CAT: 15140122), and 2.5 μg/mL amphotericin-B (Thermo Fisher Scientific, CAT: 15290026) as described previously [66]. Cells were incubated at 37°C with 5% CO_2_ and maintained at a density below 70% confluence using standard cell culture techniques. Cell passage was performed using 0.25% trypsin-ethylenediamine tetraacetic acid (EDTA, ThermoFisher, CAT: 25200056).

### Transwell^®^-based bioactivity and proliferation assay

**Figure 1.**
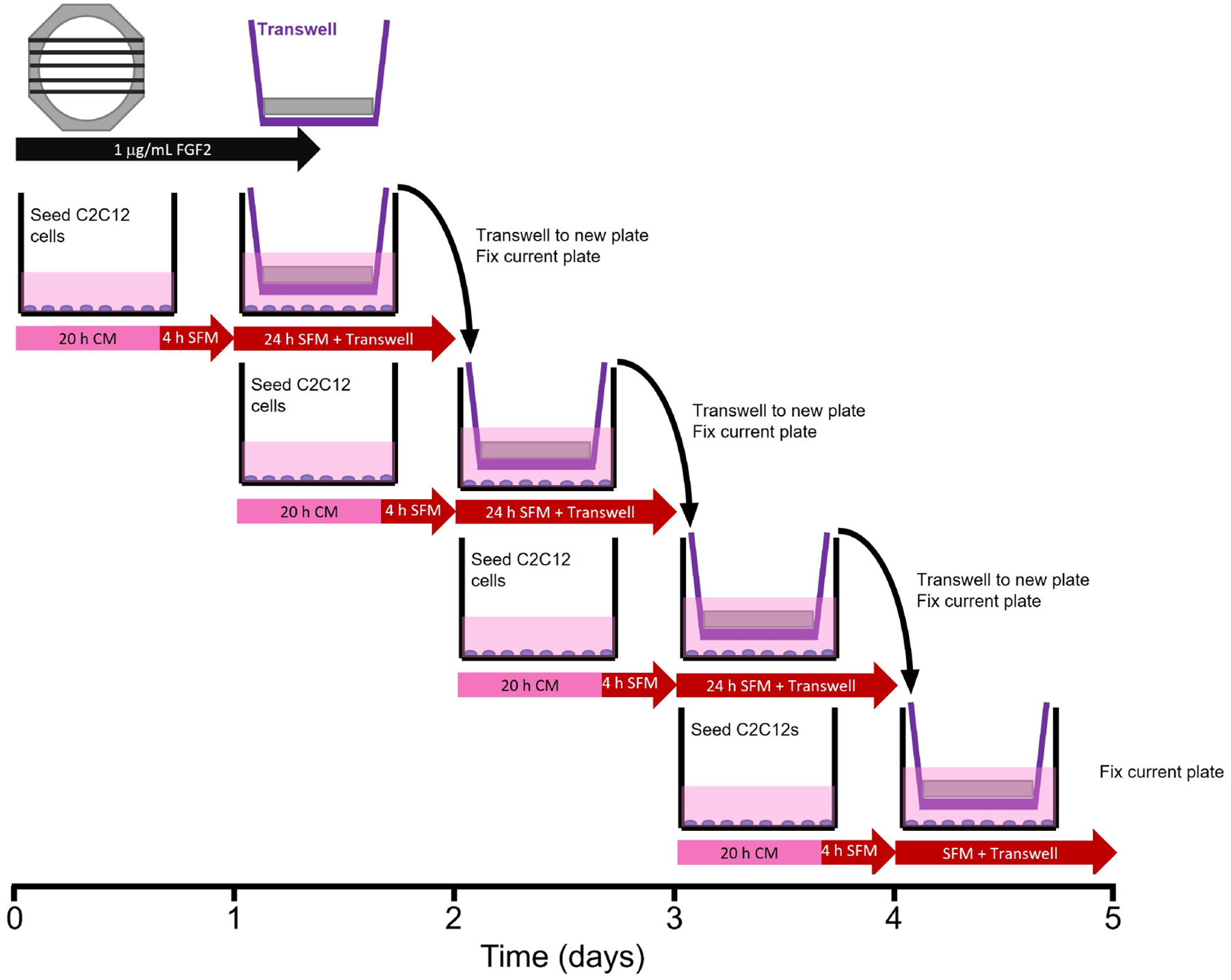
Transwell^®^-based proliferation assay. Myoblasts were seeded in 6-wells 24 h prior to the addition of the Transwell^®^ insert, which contained the microthread constructs. Medium was switched from CM to SFM 4 h prior to the addition of the Transwell^®^. After 24 h in a specific well, the Transwell^®^ insert was moved to a new well, which was seeded 24 h prior. The well from which Transwell^®^ was moved was immediately fixed with ice-cold methanol until assay completion when all wells were stained

To determine the bioactivity of FGF2 released from fibrin microthreads, a Transwell^®^-based myoblast proliferation assay was developed (Figure 1). Five distinct 1.5 cm long fibrin microthreads of the same condition were attached to PDMS rings, hydrated in PBS for 1 h, and sterilized for 2 h in 70% ethanol (Sigma-Aldrich, CAT: E7023). After sterilization microthread constructs were rinsed three times for 5 min with dH_2_O and left to dry in the biosafety cabinet overnight. Sterile microthread constructs were then blocked with 0.25% BSA for 1 h, and then loaded with 1 μg/mL sterile FGF2 in PBS for 16 h. Then, the FGF2 solution was aspirated and excess solution on microthread constructs was carefully blotted off. Microthread constructs were then placed into 6-well Transwell^®^ inserts. 50,000 C2C12 myoblasts were seeded onto the bottom of 6-well dishes in CM for 20 h. Then, the medium was removed and replaced with serum-free medium (SFM, 1:1 DMEM:F12, 100 U/mL penicillin, 100 μg/mL streptomycin, and 2.5 μg/mL amphotericin B) 4 h prior to adding the Transwell^®^ insert to the well. Once the Transwell^®^ insert was placed into the well, an additional 1 mL of SFM was added on top of the microthread construct to ensure it was fully submerged.

After the microthread-loaded Transwell^®^ insert incubated in the well plate for 24 h, it was moved to a new 6-well dish, which was seeded 24 h prior, following the same method as the previous plate. The original 6-well dish was then fixed with ice-cold methanol. This procedure continued for 4 days in culture to evaluate the effect of FGF2 release from microthreads on C2C12 proliferation as a function of time. Controls included cells that incubated in CM or in SFM, PDMS rings (no microthreads) adsorbed with FGF2, or UNX microthreads with and without adsorbed FGF2. At the end of the experiment, plates from all timepoints were permeabilized, blocked, and stained with Hoechst (Invitrogen, CAT: H3570) and a primary antibody against antigen Kiel 67 (Ki67; 1:400, D3B5, Cell Signaling Technologies, CAT: 9129). Microthreads were imaged using a fluorescent microscope (Zeiss Axiovert 200M microscope, Carl Zeiss, Model: Axiovert 200M). Nuclei were counted with ImageJ software to evaluate the percent of proliferating cells [Ki67^+^ count/Hoechst count × 100]. Normalized cell number relative to the SFM negative control was also determined by taking the Hoechst count of each condition and dividing by the SFM Hoechst count at each timepoint.

### Three-dimensional myoblast outgrowth assay

**Figure 2.**
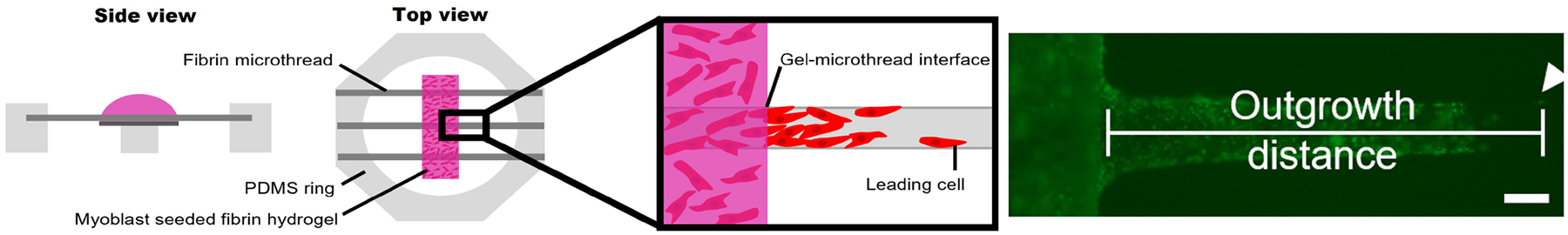
3D myoblast outgrowth assay. Side and top view of myoblast outgrowth assay, where PDMS rings anchoring three fibrin microthreads are placed on top of an elevated Thermanox coverslip, onto which a myoblast-seeded fibrin hydrogel is cast. The distance of the leading cell from the gel-microthread interface is measured over the course of four days. The representative fluorescent micrograph shows cellular outgrowth analysis on a fibrin microthread. The scale bar is 100 mm

A three-dimensional (3D) cellular outgrowth assay was performed over a period of 4 days to evaluate myoblast proliferation and migration onto fibrin microthreads, as previously described (Figure 2) [63, 78]. Briefly, rectangular Thermanox^™^ platforms (3 mm × 13 mm) were elevated 2 mm above the bottom of a 6-well with PDMS plugs (2 mm diameter) glued with medical-grade silicone adhesive. These custom plates were sterilized in 70% ethanol for 2 h, rinsed three times for 5 min with dH_2_O, and left to dry in a laminar flow hood overnight. Three distinct 1.5 cm long microthreads of the same condition were attached to PDMS rings, hydrated in dH_2_O for 1 h, sterilized in 70% ethanol for 2 h, rinsed 3 times for 5 min with dH_2_O, and left to dry in a laminar flow hood overnight. Microthreads were blocked with sterile 0.25% BSA in PBS for 1 h, then passively adsorbed with 1 mL of 0 μg/mL or 1 μg/mL sterile FGF2 in PBS for 16 h. FGF2 was aspirated, and ring constructs were carefully blotted to remove excess FGF2 solution, then transferred to the sterile 6-well dishes containing the elevated Thermanox^™^ platforms, so microthreads were laid centered and flush against the platforms. To ensure they maintained this position, rings were affixed to the bottom of 6-well plates with sterile vacuum grease.

Next, 80 μL of a myoblast-seeded fibrin gel was cast on top of each Thermanox^™^ platform. These gels were produced by mixing fibrinogen (5.22 mg/mL), CaCl_2_ (31.25 mmol/L), thrombin (3.25 U/mL), and cell solution (1,500,000 cells/mL) in an 8:1:1:2 ratio. This produced a fibrin gel with a final concentration of 3.5 mg/mL fibrinogen and a final cell concentration of 250,000 cells/mL. Prior to mixing the fibrin gel components, C2C12 myoblasts were loaded with DiI lipophilic tracer (Thermo Fisher Scientific, CAT: C7001) following the manufacturer’s instructions to facilitate tracking cellular outgrowth throughout the culture. Myoblast-populated fibrin gels were incubated at 37°C for 1 h to facilitate gel formation, then wells were flooded with 4 mL of SFM supplemented with 20 μg/mL of aprotinin to submerge the entire gel. Each condition was run in duplicate, where each replicate was considered one PDMS ring containing three microthreads of the same condition, with 6 microthread-coverslip interfaces.

To analyze cell outgrowth on the fibrin microthreads, the microthread–coverslip interfaces were imaged daily on a Zeiss Axiovert 200M fluorescent microscope, and the position of the leading cell was determined based on its distance from the edge of the thread/gel interface, as previously described (Figure 2) [63]. The medium was exchanged every 2 days. After 2 days, C2C12s were reloaded with DiI, as described previously [63]. To quantify the effect of FGF2-loaded microthreads on myoblast outgrowth, the investigators evaluated outgrowth distance and outgrowth rate. Due to regional variations in myoblast concentration throughout the fibrin hydrogel, the cell densities at the microthread-coverslip interfaces were evaluated on day 1. All microthreads with sparse initial cell densities in the hydrogel surrounding them were eliminated from further analysis. The outgrowth rate was calculated as the slope of the linear regression curve. After imaging the final day 4 timepoint, microthread constructs were fixed in 4% paraformaldehyde (Sigma-Aldrich, CAT: 158127), permeabilized in 0.1% Triton X-100 (Sigma-Aldrich, CAT: X-100), blocked with 5% BSA in PBS, and stained with Hoechst and a primary antibody against Ki67. Fluorescently labeled microthreads were imaged with a Zeiss Axiovert 200M fluorescent microscope.

### Statistical analysis

Statistical analysis was performed using Graphpad Prism 7 software (Graphpad, Software). Data was tested for normal distribution using a Shapiro-Wilk test and equal variance using Bartlett’s test. When these assumptions were met, statistical differences between conditions were determined by one-way analysis of variance (ANOVA, *P* < 0.05) with Tukey’s multiple comparisons post hoc analysis for toluidine blue, ELISA, and outgrowth data. For the Transwell^®^ proliferation assay data, a two-way ANOVA (*P* < 0.05) was performed with Tukey’s multiple comparisons post hoc analysis. Values reported are means ± standard error of the mean (SEM) unless otherwise stated. Significance is indicated as * *P* ≤ 0.05, ** *P* ≤ 0.01, *** *P* ≤ 0.001, and **** *P* ≤ 0.0001.

## Results

### Heparin can be covalently coupled to fibrin microthreads in a dose-dependent manner

**Figure 3.**
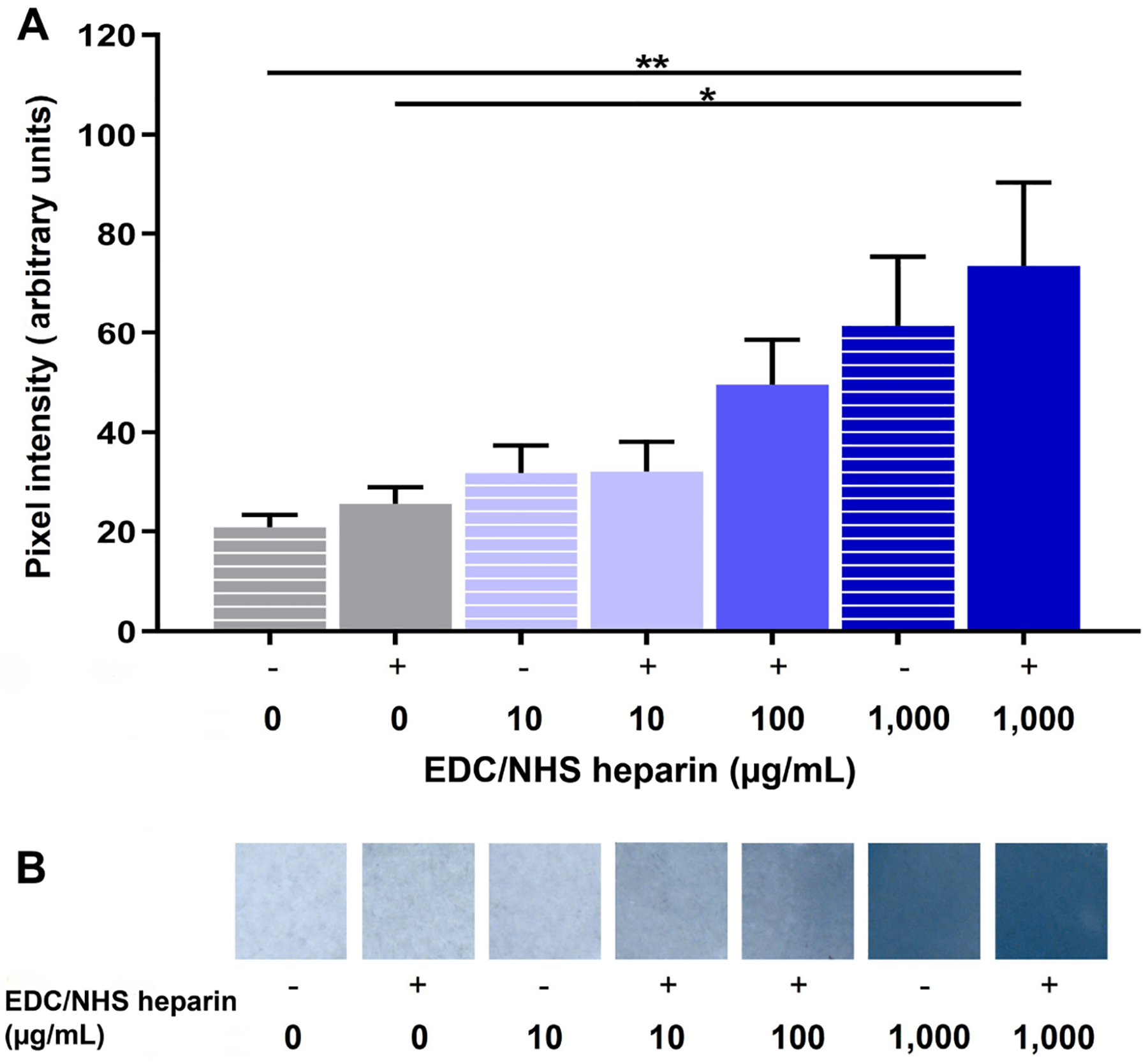
Toluidine blue analysis of heparin-conjugated fibrin films. (A) Pixel intensity analysis of UNX passively adsorbed (EDC−) and covalently conjugated (EDC+) fibrin films with varying heparin concentrations stained with toluidine blue; (B) stereo microscope images of toluidine blue dyed fibrin films, where the blue dye uptake increases with increasing heparin concentration. Statistical significance is indicated by * *P* < 0.05 and ** *P* < 0.01 between corresponding groups determined by one-way ANOVA with Tukey’s multiple comparisons post hoc analysis (*N* ≥ 3 experimental replicates)

**Figure 4.**
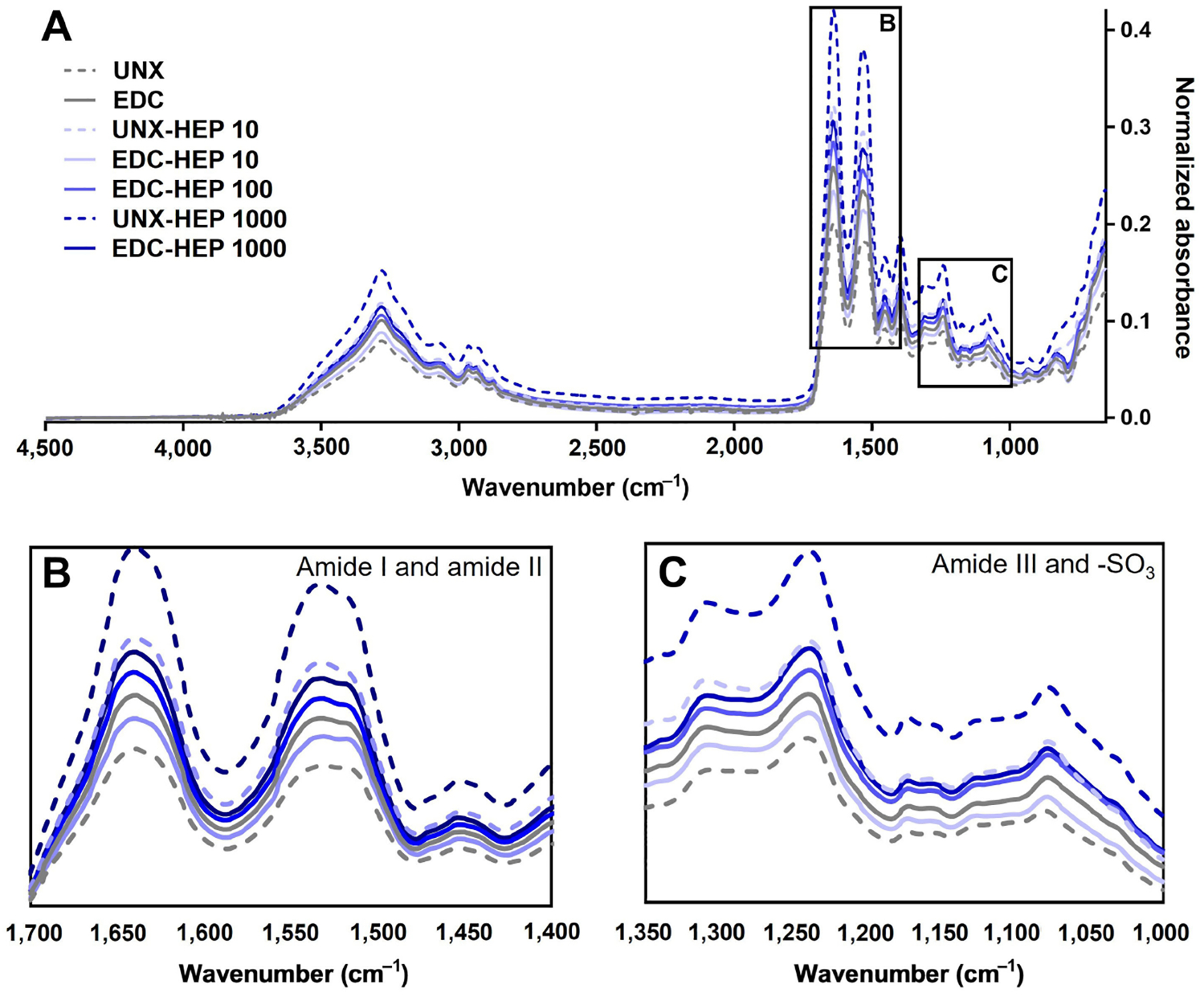
FTIR analysis of heparin-conjugated fibrin films. (A) Representative FTIR spectra of fibrin films passively adsorbed or covalently conjugated with heparin; (B) spectral regions of amide I and amide II; (C) spectral regions of amide III and sulfonated groups (−SO_3_). Representative data from *N* ≥ 3 experimental replicates

To create a conjugation strategy that mimics the native sequestration of FGF2 by heparan sulfate proteoglycans in the basement membrane, we covalently conjugated heparin to the surface of fibrin microthread and film-based scaffolds. This was done by employing carbodiimide chemistry, which covalently reacted the free amine groups of fibrins with the activated carboxylic acid groups on heparin. The dose-dependent conjugation of heparin to fibrin microthreads was evaluated with toluidine blue staining (Figure 3) and FTIR (Figure 4). Toluidine blue staining of fibrin films showed an increase in dye uptake with increasing heparin concentration, as anticipated (Figure 3). The same trend was observed when toluidine blue staining was performed on heparin-conjugated fibrin microthreads (data not shown). Pixel intensity analyses of images were performed to further quantify this finding on fibrin films (Figure 3). Pixel intensity quantification demonstrates increasing dye uptake with increasing heparin concentration. The highest concentration of heparin evaluated, EDC-HEP 1000 films, had significantly higher pixel intensity compared to UNX (*P* ≤ 0.01) and EDC (*P* ≤ 0.05) films. No statistically significant differences were observed between UNX and EDC films with the same heparin concentrations, indicating that the EDC crosslinking reaction did not increase heparin binding to fibrin microthreads compared to corresponding passively adsorbed UNX conditions.

To further analyze the binding of heparin to fibrin scaffolds, FTIR analysis was performed to analyze changes in secondary structure as a result of heparin incorporation (Figure 4A). Heparin adsorption was characterized by the formation of amide bonds and analyzed by amide I, amide II, and amide III peaks centered at 1,630 cm^−1^, 1,520 cm^−1^, and 1,240 cm^−1^, respectively. All scaffold conditions covalently coupled with heparin appeared to have higher amide peaks than UNX scaffolds (Figure 4B and C). The adsorption band at 1,240 cm^−1^ is also indicative of the presence of sulfonated groups (-SO_3_ stretching), which suggests the presence of heparin as it is a sulfated polysaccharide (Figure 4C) [77]. Taken together, these data suggest the incorporation of heparin in fibrin films, despite challenges in differentiating variations in overlapping peaks.

### Sustained release of FGF2 from heparin conjugated and co-inc microthreads

**Figure 5.**
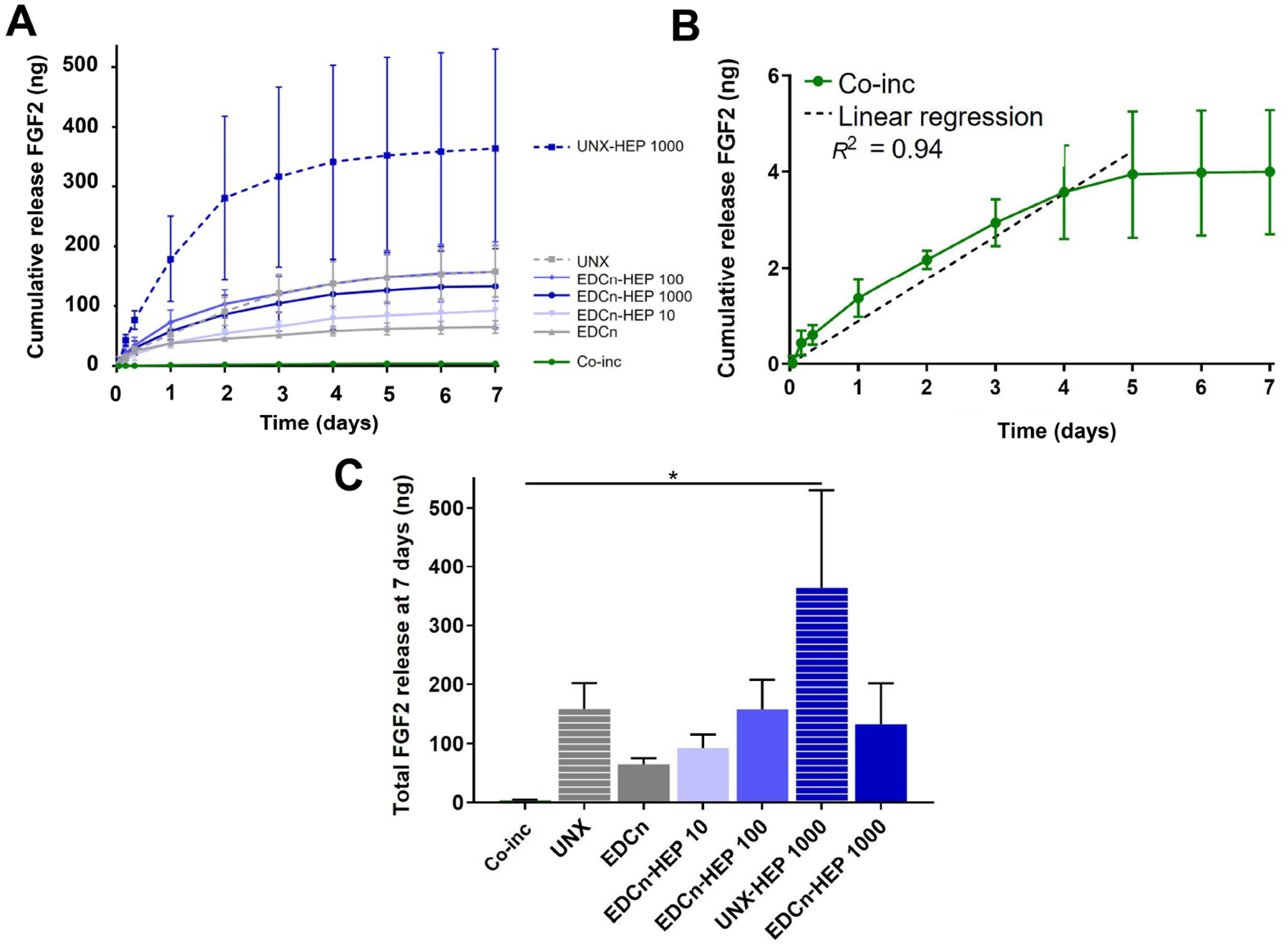
FGF2 release kinetics from fibrin microthreads. (A) Cumulative sustained release of FGF2 over one week from heparin conjugated and co-inc fibrin microthreads; (B) linear regression of FGF2 release from co-inc microthreads through day five (dashed line) showed that co-inc scaffolds achieved zero-order release kinetics of FGF2 (*R*^2^ = 0.94); (C) total FGF2 released from fibrin microthreads after one week reveals that UNX-HEP 1000 microthreads had the highest total FGF2 release at seven days. Statistical significance is indicated by * (*P* < 0.05) between corresponding groups determined by one-way ANOVA with Tukey’s multiple comparisons post hoc analysis (*N* ≥ 3 experimental replicates). EDCn: microthreads EDC crosslinked in neutral buffer

Cumulative release kinetics of FGF2 from heparin-conjugated and co-inc fibrin microthreads were evaluated over a period of 7 days with an ELISA. Co-inc fibrin microthreads demonstrated sustained release of FGF2 over the course of five days, at which point the scaffolds were largely degraded and FGF2 release diminished (Figure 5A and B). A linear regression analysis of co-inc microthread release kinetics through 5 days, constrained through the origin, showed that co-inc scaffolds achieved zero-order release kinetics of FGF2 (*R*^2^ = 0.94) (Figure 5B). Co-inc microthreads had the lowest total quantity of FGF2 release compared to passively adsorbed microthread conditions (Figure 5C).

Fibrin microthreads passively adsorbed with FGF2 also yielded sustained release of FGF2 over the course of one week (Figure 5A). Fibrin microthreads covalently conjugated with heparin via EDC crosslinking appeared to have a higher cumulative release compared to EDC crosslinked threads, although this finding is not statistically significant (Figure 5C). EDC-HEP 100 and EDC-HEP 1000 had similar release profiles, with similar total FGF2 release. Interestingly, UNX microthreads also yielded sustained release of FGF2 over the course of one week, with an average total release comparable to EDC-HEP 100 and EDC-HEP 1000 microthreads (Figure 5A and C). Microthreads passively adsorbed with heparin appeared to have higher total FGF2 release compared to microthreads carbodiimide conjugated with heparin at the same concentration. UNX-HEP 1000 microthreads released 2.75-fold more FGF2 than EDC-HEP 1000 microthreads. UNX-HEP 1000 released a total of 363 ng ± 289 ng FGF2, while EDC-HEP 1000 microthreads released 133 ng ± 120 ng FGF2. UNX-HEP 1000 microthreads also appeared to have a higher initial burst release, where 77% of total FGF2 was released within the first two days, compared to 65% and 64% released from EDC-HEP 100 and EDC-HEP 1000 microthreads at two days, respectively.

### FGF2 released from fibrin microthreads remains bioactive and stimulates myoblast proliferation

**Figure 6.**
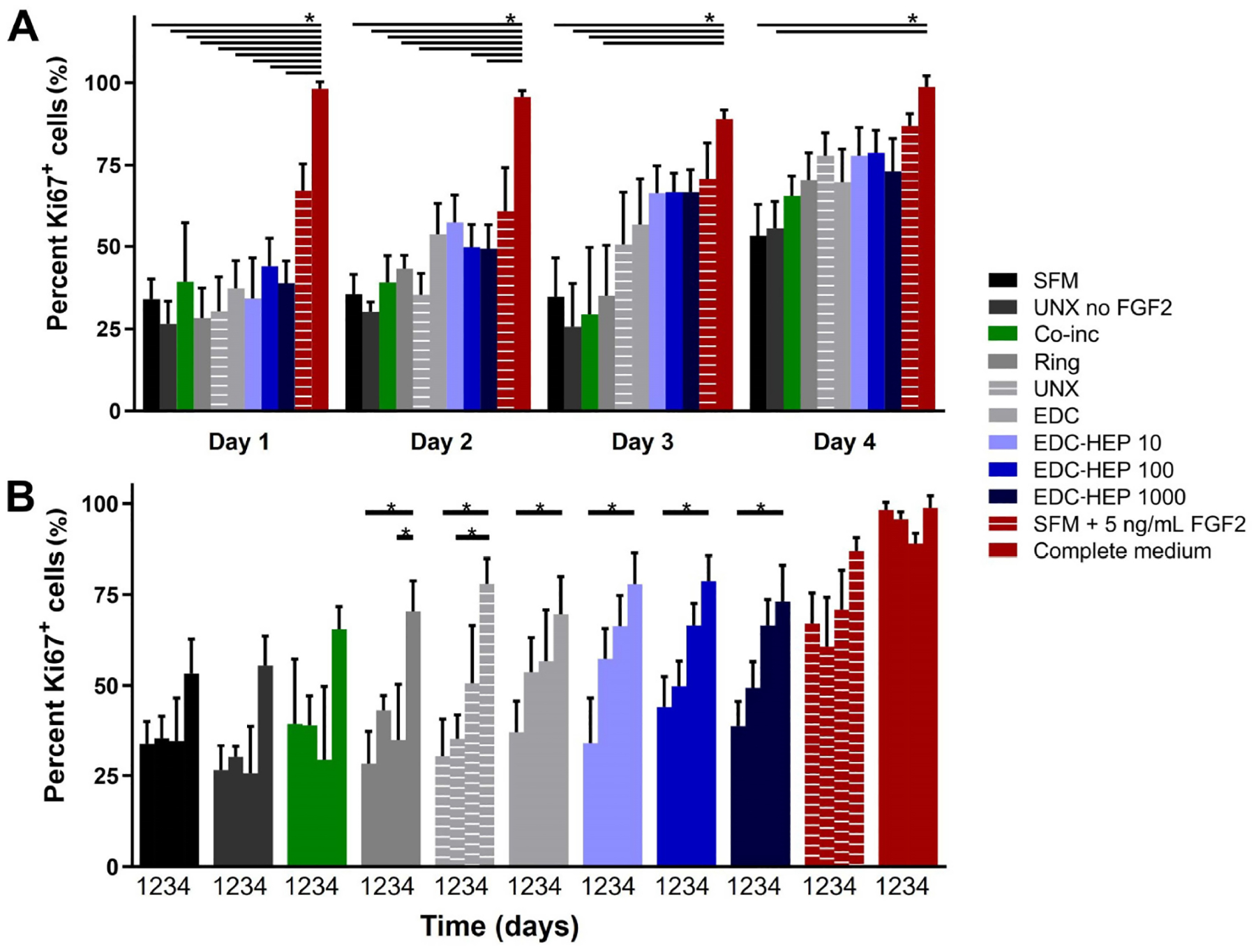
Transwell^®^-based proliferation assay to determine FGF2 bioactivity and its effect on *in vitro* percent Ki67^+^ myoblasts. (A) Percentage of Ki67^+^ cells is presented as a function of time and demonstrates trends in increasing percent Ki67^+^ cells with fibrin microthreads passively adsorbed with FGF2; (B) the same data presented as a function of FGF2 incorporation strategy demonstrates that the percent Ki67^+^ myoblasts significantly increase with time for all conditions passively adsorbed with FGF2. Statistical significance is indicated by * (*P* < 0.05) between corresponding groups determined by two-way ANOVA with Tukey’s multiple comparisons post hoc analysis (*N* ≥ 3 experimental replicates)

A Transwell^®^-based proliferation assay was performed to determine the bioactivity of FGF2 released from fibrin microthreads over the course of four days. Fibrin microthread constructs in Transwell^®^ inserts were placed in 6-well plates seeded with myoblasts cultured in SFM for 24 h before being moved to a new myoblast-seeded well (Figure 1). Plates for each timepoint were fixed and stained with proliferation marker Ki67 to evaluate the effect of FGF2 released from fibrin microthreads on myoblast proliferation over time. At each timepoint, there was no statistically significant difference in the percent of Ki67^+^ myoblasts between FGF2-loaded fibrin microthread conditions (Figure 6A). FGF2-loaded fibrin microthreads appeared to stimulate higher percent Ki67^+^ myoblasts on days 2–4 compared to SFM and UNX no FGF2 control conditions, although this trend was not statistically significant (Figure 6A). The cell proliferation data was also analyzed by looking at each condition as a function of time (Figure 6B). The percentage of Ki67^+^ myoblasts significantly increased from day 1 to day 4 in all microthread conditions passively adsorbed with FGF2, including UNX, EDC, and EDC-HEP 10, EDC-HEP 100, and EDC-HEP 1000 microthreads (Figure 6B).

**Figure 7.**
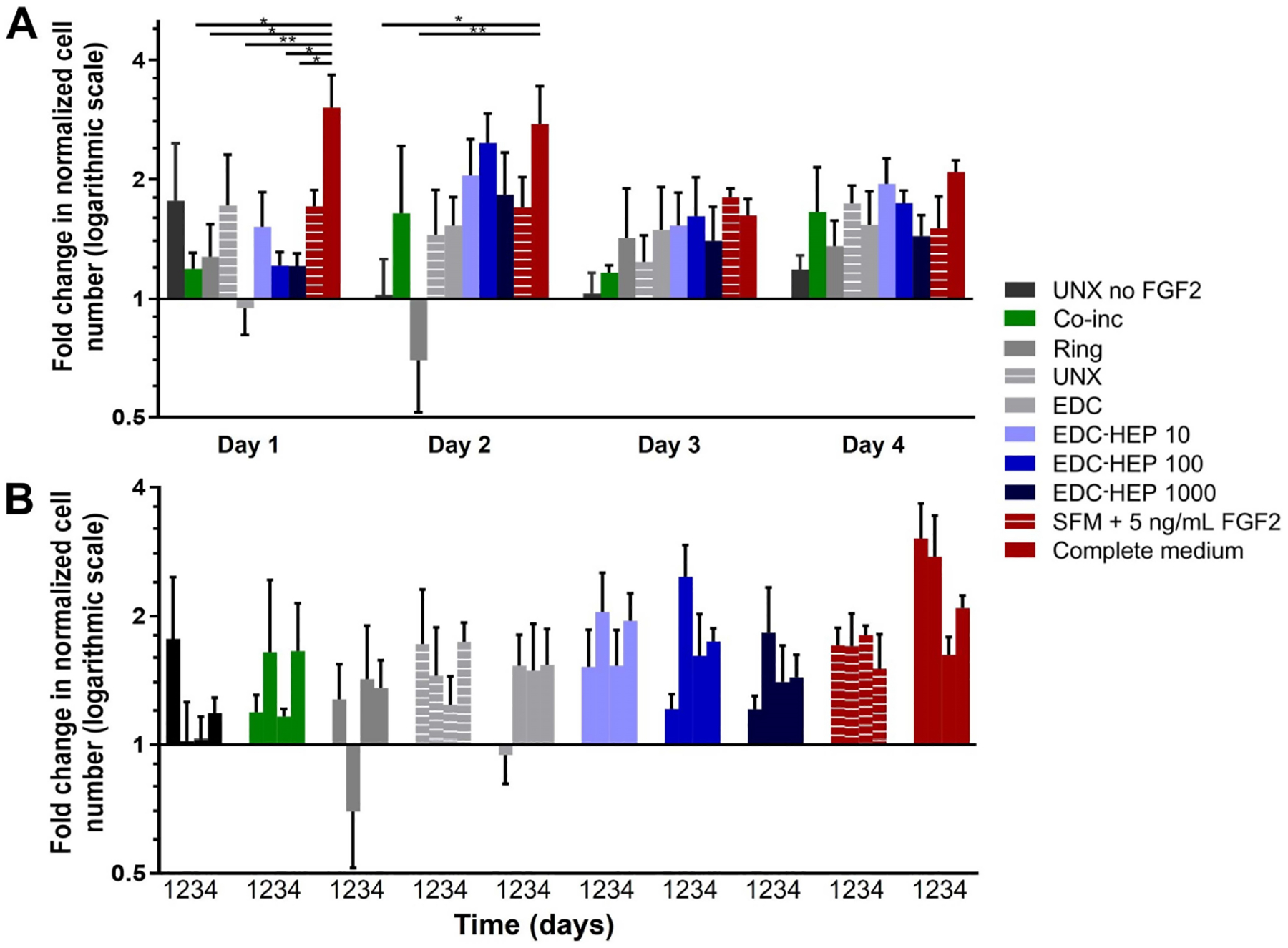
Transwell^®^-based proliferation assay to determine FGF2 bioactivity and its effect on *in vitro* myoblast normalized cell number. (A) Fold change in cell number normalized to SFM cell number as a function of time; (B) the same data presented as a function of FGF2 incorporation strategy indicate increased cell numbers in all FGF2 loaded microthreads. Statistical significance is indicated by * (*P* < 0.05) between corresponding groups determined by two-way ANOVA with Tukey’s multiple comparisons post hoc analysis (*N* ≥ 3 experimental replicates)

Further analyses were conducted to evaluate myoblast number normalized to the SFM negative control (Figure 7). At each time point, all microthreads loaded with FGF2, including co-inc and EDC-HEP microthreads, had elevated cell numbers relative to the SFM negative control condition (Figure 7A). Additionally, normalized myoblast cell numbers remained elevated over the course of four days for FGF2 co-inc and EDC-HEP microthreads (Figure 7B). Taken together with the Ki67 data, these data demonstrate that FGF2 co-inc within or passively adsorbed to fibrin microthreads is released from the scaffolds over time and acts as a mitogen to stimulate myoblast proliferation.

### Myoblast outgrowth on FGF2-loaded fibrin microthreads

**Figure 8.**
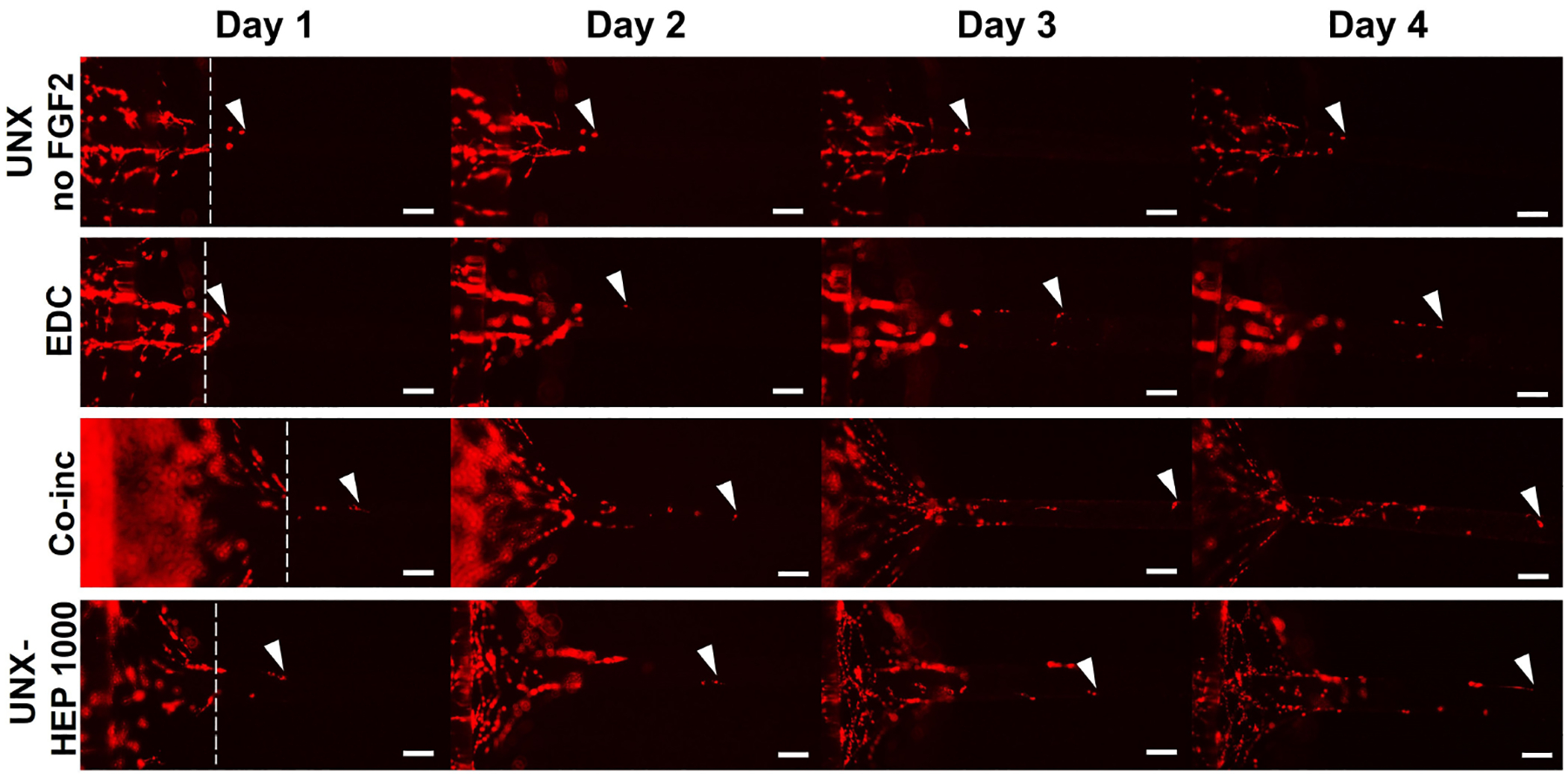
Representative images of myoblast outgrowth on fibrin microthreads. Outgrowth on fibrin microthreads with no FGF2 (UNX no FGF2), co-inc with FGF2 (co-inc), or passively adsorbed with FGF2 (EDC, UNX-HEP 1000). Myoblast outgrowth (visualized with DiI staining) was observed as several “leading” myoblasts furthest out on the microthread, which was followed by a more confluent layer of cells. White dotted lines show the microthread-gel interface on day one. White arrows indicate the leading cell position. The scale bar is 100 μm

**Figure 9.**
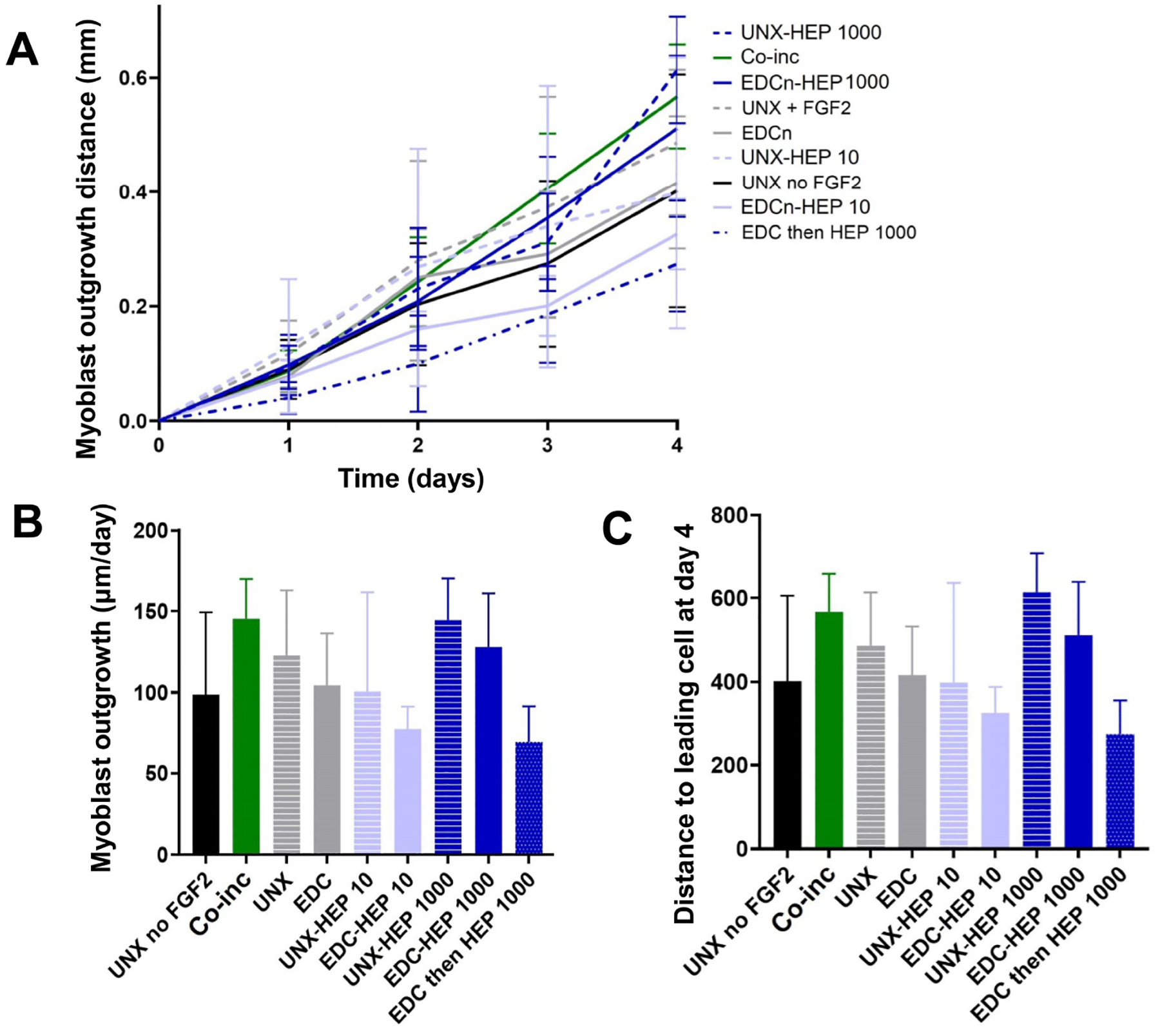
Myoblast outgrowth on FGF2-loaded fibrin microthreads. (A) C2C12 myoblast outgrowth distance on fibrin microthreads as a function of time. Linear regression analysis constrained through the origin revealed that myoblast outgrowth rate was linear on all microthread conditions (0.92 < *R*^2^ < 0.99); (B) myoblast outgrowth was calculated as the linear slope over a period of four days and indicates that myoblasts on co-inc and UNX-HEP 1000 microthreads had the highest rate of outgrowth; (C) similar trends were observed when evaluating total distance traveled of the leading cell at day four (*N* ≥ 3 experimental replicates)

A cellular outgrowth assay was performed to determine the effect of FGF2-loaded fibrin microthreads on myoblast proliferation and migration over the course of four days. A 3D outgrowth assay was used, where a myoblast-seeded fibrin hydrogel is cast over suspended fibrin microthreads so myoblasts can proliferate and migrate onto the microthreads over several days (Figure 2). For most conditions, outgrowth was observed as several “leading” myoblasts on the microthread, which was followed by a more confluent layer of cells (Figure 8). All fibrin microthread conditions evaluated demonstrated a linear outgrowth rate, where outgrowth increased over time (Figure 9A). A linear regression analysis of myoblast outgrowth kinetics through day four constrained through the origin demonstrated that myoblast outgrowth rate was linear on all microthread conditions (0.92 < *R*^2^ < 0.99). Myoblast outgrowth rate was highest on co-inc and UNX-HEP 1000 microthreads, which both had a rate of 145 μm/day (Figure 9B). This is approximately 1.5-fold higher than the rate of myoblast outgrowth observed on UNX microthreads with no FGF2. When comparing microthread conditions loaded with 1,000 μg/mL of heparin, UNX-HEP 1000 microthreads had the highest outgrowth rate, followed by EDC-HEP 1000, and finally EDC then HEP 1000. Similar trends between conditions were observed when evaluating the distance of the leading cell on day four (Figure 9C). Myoblasts traveled an average of 273–614 μm over the course of four days in culture, depending on microthread condition.

**Figure 10.**
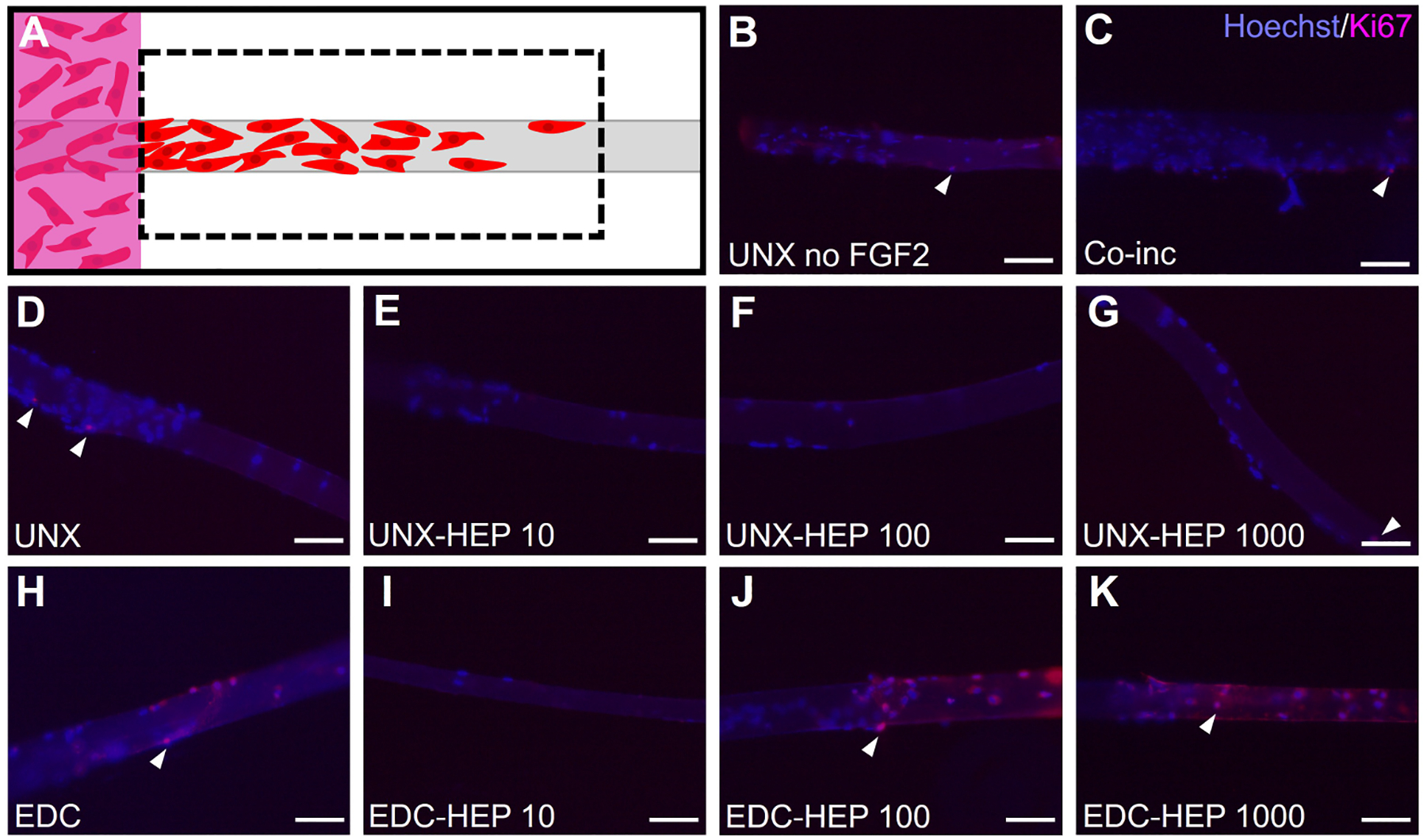
Evaluating myoblast proliferation on fibrin microthreads from the 3D outgrowth assay. (A) At the terminal day four timepoint of the myoblast outgrowth assay, microthreads were fixed, removed from outgrowth assays, and stained with Hoechst and Ki67 to determine the extent of myoblast proliferation; (B) Ki67 stained microthreads representative image of UNX with no FGF2; (C) Ki67 stained microthreads representative image of microthreads co-inc with 1 μg/mL F1GF2; (D) Ki67 stained microthreads representative image of UNX microthreads co-inc with 0 μg/mL heparin and passively adsorbed with 1 μg/mL FGF2; (E) Ki67 stained microthreads representative image of UNX microthreads co-inc with 10 μg/mL heparin and passively adsorbed with 1 μg/mL FGF2; (F) Ki67 stained microthreads representative image of UNX microthreads co-inc with 100 μg/mL heparin and passively adsorbed with 1 μg/mL FGF2; (G) Ki67 stained microthreads representative image of UNX microthreads co-inc with 1,000 μg/mL heparin and passively adsorbed with 1 μg/mL FGF2; (H) Ki67 stained microthreads representative image of EDC crosslinked microthreads co-inc with 0 μg/mL heparin and passively adsorbed with 1 μg/mL FGF2; (I) Ki67 stained microthreads representative image of EDC crosslinked microthreads co-inc with 10 μg/mL heparin and passively adsorbed with 1 μg/mL FGF2; (J) Ki67 stained microthreads representative image of EDC crosslinked microthreads co-inc with 100 μg/mL heparin and passively adsorbed with 1 μg/mL FGF2; (K) Ki67 stained microthreads representative image of EDC crosslinked microthreads co-inc with 1,000 μg/mL heparin and passively adsorbed with 1 μg/mL FGF2. The scale bar is 100 μm

Myoblast outgrowth is known to be a function of both proliferation and migration [63, 78]. To uncouple the cellular mechanism primarily contributing to the myoblast outgrowth observed on FGF2-loaded fibrin microthreads, the investigators performed Ki67 staining of fibrin microthreads from the 3D myoblast outgrowth assay at the terminal day four timepoint by fixing and removing microthreads from the assay to stain and image (Figure 10A). Representative images of Ki67-stained fibrin microthreads display minimal Ki67 staining at four days in conditions with no FGF2, co-inc with FGF2, and passively adsorbed with FGF2 (Figure 10B–K). As such, this result was not further quantified.

## Discussion

This study focused on the development of fibrin microthread scaffolds with the sustained, physiologically relevant release of FGF2, with the goal of developing an implantable scaffold to treat VML injuries. To generate fibrin microthreads with sustained release of FGF2, this study evaluated two strategies to incorporate the growth factor. First, we covalently conjugated heparin to fibrin microthreads via carbodiimide coupling, creating a biomimetic strategy mimicking FGF2 sequestration in native skeletal muscle ECM (EDC-HEP). Alternatively, we incorporated FGF2 within fibrin microthreads by mixing FGF2 with fibrinogen prior to co-extrusion (co-inc). Fibrin is ideally suited for incorporating FGF2 because it binds with high affinity to FGF2 and protects it from proteolytic degradation [68]. Toluidine blue staining and FTIR confirmed heparin conjugation to fibrin microthreads by demonstrating increasing dye uptake and amide bond peaks, respectively. FGF2 release kinetics revealed that fibrin microthreads conjugated with heparin had sustained release over one week and delivered a higher total amount of FGF2 than EDC microthreads without heparin. Additionally, microthreads co-inc with FGF2 achieved zero-order release kinetics over five days. UNX-HEP 1000 microthreads had the highest total FGF2 release but appeared to have a greater initial burst release compared to EDC-HEP 1000 microthreads, which may be due to the non-covalent attachment of heparin. A Transwell^®^-based proliferation assay demonstrated that FGF2 released from fibrin microthread scaffolds was bioactive, stimulating myoblast proliferation over a period of four days *in vitro*. Finally, a 3D outgrowth assay demonstrated that co-inc and heparinconjugated microthreads may enhance myoblast outgrowth. Minimal Ki67^+^ myoblasts on these microthreads suggest that outgrowth is primarily driven by cellular migration. Taken together, these results suggest that heparinconjugated fibrin microthreads create a biomimetic delivery strategy for FGF2 with the added benefit of providing mechanical strength and anisotropic alignment cues from a fiber-based scaffold. This work addresses limitations in the field to develop a scaffold that synergistically provides biochemical and biophysical cues simultaneously. We anticipate that the combined effects of fibrin microthread mechanical properties, topographic alignment cues, and the therapeutic release of FGF2 will facilitate a promising scaffold strategy for enhancing functional muscle regeneration following VML injuries.

We implemented a biomimetic FGF2 delivery approach by passively adsorbing or chemically immobilizing heparin sodium salt to fibrin microthreads. In passively adsorbed conditions, heparin likely binds to fibrin primarily through electrostatic interactions, as well as the hydrophobic effect and hydrogen bonding [79]. Carbodiimide crosslinking was employed to create a covalent amide bond between the carboxyl groups of heparin and the amine groups of fibrin. We chose to utilize carbodiimide crosslinking because it creates a stable covalent bond and is a “zero-length” crosslinker, meaning that no residues from the crosslinking reaction become a part of the bond structure [80]. Previous studies demonstrated that EDC crosslinking chemistry can be used to covalently couple heparin to polymer scaffolds with free amine groups, including collagen [32, 80–82], fibrin [43, 83], poly-lactic-co-glycolic acid (PLGA) [84], and poly(ε-caprolactone) (PCL) [73]. Toluidine blue staining and FTIR confirmed that heparin was successfully conjugated to fibrin microthreads in a dose-dependent manner. Both passively adsorbed (UNX-HEP) and covalently conjugated (EDC-HEP) conditions demonstrated increasing toluidine blue dye uptake and amide bond peaks with increasing concentrations of heparin from 0 μg/mL to 1,000 μg/mL. Researchers previously demonstrated an increasing quantity of heparin conjugated to collagen scaffolds by increasing the concentration of heparin within the crosslinking solution, which was determined by a toluidine blue or dimethylmethylene blue assay [80, 82].

In the future, additional methods to modify the degree of heparin conjugation to fibrin microthreads could be investigated. This could include altering the EDC crosslinking reaction time and pH, which have both been shown to affect the degree of heparin conjugation to polymer scaffolds [73, 80]. Despite being an effective conjugation strategy, EDC crosslinking can yield scaffolds that are largely resistant to proteolytic degradation [18]. Upon implantation into a hind limb murine VML defect, EDC crosslinked fibrin microthreads persisted through 60 days post-implantation [18]. Ideally, these scaffolds should degrade at the same rate as new tissue infiltration, so it can provide provisional structural support yet ultimately be replaced by functional tissue. Alternative, more sophisticated heparin conjugation strategies have also been investigated [35, 85–88]. One study covalently immobilized a bi-domain peptide to fibrin through transglutaminase activity, and then bound heparin to this peptide through non-covalent, electrostatic interactions [35]. This release system was able to sustain the release of FGF2 from heparinized fibrin matrices, improving dorsal root ganglia neurite extension *in vitro* [87]. Finally, the development of synthetic heparin mimetics eliminates concerns with heparin’s inherent heterogeneity and allows for precise control over structure and binding affinity [79]. Maynard and Hubbell [88] developed a tetrapeptide of sulfated amino acids, which bound VEGF with high affinity. Freeman et al. [89] mimicked heparin binding by sulfating the uronic acid groups in alginate and hyaluronan (HA), based on the knowledge that the degree of heparin sulfation influences growth factor binding [90, 91]. They found that sulfated alginate and HA provided strong binding and sustained release of heparin-binding growth factors such as FGF2 [89]. Ultimately, tuning of the EDC covalent crosslinking conjugation strategy, or developing more sophisticated linker peptides or heparin mimetics may allow for further tuning of heparin conjugation to fibrin microthread scaffolds.

Co-inc fibrin microthreads yielded sustained release of FGF2 over the course of five days. After a muscle crush injury, FGF2 is detected in wound fluid 2–8 days after injury and peaks at 6–8 days [30]. This emphasizes the need for sustained release of FGF2 over the course of one week to mimic its *in vivo* temporal release. Previous work in our laboratory initially developed this co-incorporation strategy and found that FGF2 was well distributed throughout the fibrin microthreads, but did not evaluate its release kinetics from the scaffold [70]. We found that co-inc microthreads had sustained release of FGF2 over five days, at which point the scaffolds were largely degraded and FGF2 release attenuated. FGF2 binds with high affinity to fibrin and protects it from proteolytic degradation [68], which we hypothesize enables the scaffolds to achieve sustained release. A linear regression analysis of release kinetics through day five revealed that co-inc scaffolds achieved zero-order release kinetics of FGF2 (*R*^2^ = 0.94). Jeon et al. [84] also achieved zero-order release of FGF2 from heparin-conjugated PLGA microspheres encapsulated within a fibrin hydrogel. They found that zero-order release kinetics of FGF2 were achieved over four weeks by increasing the fibrinogen concentration to 94 mg/mL or higher. Fibrin microthread scaffolds are analogous to dense hydrogels as they also have a high fibrinogen concentration (35 mg/mL), which may explain how they mediate the controlled release behavior observed. Because these scaffolds were not crosslinked, it was hypothesized that release was mediated by a combination of hydrolysis, bulk degradation, dissociation of FGF2 from fibrin, and diffusion through the microthread. Co-inc microthreads also had the smallest total quantity of FGF2 release which is likely explained by differences in loading strategies.

Fibrin microthreads covalently conjugated with heparin yielded sustained release of FGF2 over the course of one week, whereas higher heparin conjugation resulted in a more sustained release and a higher total amount of FGF2 released. This result is similar to work by other researchers who conjugated heparin to polymer scaffolds to mediate the release of FGF2 [43, 84, 92] and other heparin-binding growth factors [82, 83, 93, 94]. Younesi et al. [82] also showed that increasing the concentration of heparin in the EDC crosslinker bath from 0 mg/mL to 10 mg/mL created a more sustained release of platelet-derived growth factor (PDGF) from collagen microthreads over the course of 15 days, with the majority of release taking place in the first week. Yang et al. [43] observed similar sustained release of FGF2 from a fibrinogen scaffold conjugated with heparin via EDC crosslinking. They found only 40% of FGF2 released from their scaffold by one week, and sustained release of FGF2 continued for up to three weeks. Because this study utilized the same scaffold biomaterial (fibrin), heparin conjugation strategy (EDC), and growth factor (FGF2) as this work, we hypothesize it is very likely that FGF2 remains bound to heparin-conjugated fibrin microthreads after one week. This would be expected, as the electrostatic interaction between FGF2 and heparin is a strong noncovalent bond [79]. We performed a glycine rinse to prevent any residual activated carboxylic acid groups from the EDC reaction from covalently binding FGF2 to fibrin [71, 72]. FGF2 release from heparin-conjugated fibrin microthreads likely occurs through different mechanisms, including FGF2 dissociation from heparin and degradation of fibrin [35, 92]. *In vivo*, additional factors would influence FGF2 release including proteolytic degradation of fibrin via plasmin and enzymatic degradation of heparin via heparinase [35], which likely leads to faster release of FGF2 than what is observed *in vitro*. Future analyses to evaluate the extended release of FGF2 over several weeks and to quantify FGF2 remaining on our scaffolds will provide more information about how heparin-conjugated fibrin microthreads mediate FGF2 binding, sequestration, and release.

Interestingly, UNX and UNX-HEP 1000 microthreads both yielded higher total FGF2 release compared to EDC microthreads crosslinked with the same corresponding heparin concentration (EDC and EDC-HEP 1000, respectively). UNX-HEP 1000 microthreads also exhibited a higher initial burst release, where 77% of total FGF2 was released within the first two days, compared to 65% and 64% of total FGF2 released from EDC-HEP 100 and EDC-HEP 1000 microthreads at two days, respectively. This is a similar finding to the work by Yang et al. [43], who compared FGF2 release from fibrin hydrogels containing free and EDC-conjugated heparin. They found that fibrin hydrogels containing free heparin had less sustained release than heparin conjugated fibrinogen, and exhibited a much higher initial burst release within the first few days [43]. This is likely because electrostatic attraction forces in fibrin with free heparin are weaker than the covalent bond formed when heparin is carbodiimide crosslinked to fibrin. The high sustained release from UNX microthreads may be due to the high binding affinity FGF2 has for fibrin, with reported equilibrium dissociation constant (K_d_) ranging from 0.8–261 nmol/L [68]. Thus, it was hypothesized that FGF2 binds with specificity to saturate UNX microthreads, which mediates its sustained release from the scaffold. Visual inspection indicated that UNX and UNX-HEP 1000 microthreads were largely degraded after the one-week release assay, so it is likely that the total FGF2 release reported is close to the total amount of FGF2 initially loaded on these scaffolds. In contrast, EDC microthreads had not noticeably degraded at the terminal one-week timepoint of the release assay.

We hypothesize that differences in cumulative release between fibrin microthread conditions are likely a result of differences in initial FGF2 binding as well as the release mechanisms. Researchers demonstrated that increasing concentrations of heparin when EDC crosslinked to collagen scaffolds is correlated with an increase in FGF2 binding [74, 95, 96]. A 2-fold to 3.4-fold increase in the amount of bound FGF2 was observed on heparinized collagen matrices compared to unmodified collagen scaffolds, as determined by measuring the radioactivity of 125 iodinated (125I)-FGF2 [74, 95, 96]. Higher FGF2 binding was also observed on heparinized, EDC-crosslinked PLGA nanofibers compared to unmodified nanofibers [86]. Based on these studies, the increasing concentrations of heparin increased the amount of FGF2 binding to fibrin microthreads. It is not clear whether the effect of passively adsorbing *vs*. chemically conjugating heparin would influence the efficiency of FGF2 binding. Quantification of toluidine blue staining did not reveal any significant differences in heparin incorporation on passively adsorbed *vs*. chemically conjugated scaffolds with the same heparin concentration. Because of this, we hypothesize that FGF2 binding is comparable on passively adsorbed and EDC-coupled scaffolds when heparin concentration is held constant. This should be investigated in future studies following complete scaffold digestion.

FGF2 incorporated onto or within fibrin microthreads remained bioactive, as demonstrated by its ability to stimulate myoblast proliferation in a Transwell^®^-based assay. FGF2 is a known mitogen for myoblasts [26–29, 97, 98]. FGF2-loaded microthreads stimulated myoblast proliferation comparable to 5 ng/mL FGF2-supplemented SFM. Additionally, the percent of Ki67^+^ myoblasts increased significantly from day 1 to 4 in all microthread conditions passively adsorbed with FGF2. These results further confirmed that (1) co-inc and heparin-mediated FGF2 incorporation strategies did not inhibit the bioactivity of FGF2, and (2) FGF2 released from heparin-conjugated microthreads upregulated myoblast function over a prolonged time. Other researchers performed similar bioactivity assays by placing FGF2-loaded scaffolds into Transwell^®^ culture inserts and evaluating the effect of FGF2 release on fibroblast [84, 92] or endothelial cell [43] number over time [43, 84]. These studies also showed that released FGF2 remained bioactive, as it stimulated proliferation to the same degree as the FGF2-supplemented medium. The Transwell^®^-based proliferation assay relies on bound FGF2 releasing from fibrin microthreads and diffusing through the Transwell^®^ membrane to stimulate myoblast proliferation. To assess the ability of bound FGF2 on fibrin microthreads to stimulate myoblast proliferation and migration, we performed a 3D cellular outgrowth assay.

Our lab developed a 3D cellular outgrowth assay, an *in vitro* model system that examines the combined effect of cellular proliferation and migration, termed outgrowth, onto fibrin microthreads from a myoblast-populated hydrogel [63, 78]. This assay more accurately recapitulates myoblast outgrowth from a wound margin compared to other commonly used migration assays including Transwell^®^ inserts and scratch assays [99]. The outgrowth assay enables the evaluation of the proliferation and migration of myoblasts directly attached to the microthreads; this allows us to measure the influence of the bound heparin and FGF2 complex, where other migration assays rely on FGF2 being released from the scaffold. This is particularly important for analyzing heparin-conjugated scaffolds because they likely sequester more FGF2, and bound heparin facilitates FGF2 binding with its receptor [45, 46, 100].

FGF2-loaded fibrin microthreads appeared to influence myoblast outgrowth as a function of the growth factor incorporation strategy. Outgrowth rates were highest on co-inc and UNX-HEP 1000 microthreads, which were both approximately 1.5-fold higher than the rate of myoblast outgrowth observed on UNX microthreads with no FGF2. Similarly, Cornwell and Pins [70] saw an approximately 2-fold increase in fibroblast outgrowth on fibrin microthreads co-inc with 200 ng/mL FGF2. However, this assay was carried out in proliferation media with 2% FBS, which is more conducive to cellular proliferation and migration than the SFM used in the present study. Although not significant, there appeared to be a trend where EDC crosslinked microthreads directed lower myoblast outgrowth rates and distances compared to UNX microthreads with corresponding heparin concentrations. Researchers demonstrated that EDC crosslinking of collagen films reduced C2C12 binding and spreading, which they hypothesize was caused by a reduction in the number of available cell binding sites [101]. Bax et al. [102] demonstrated that with an increasing degree of EDC crosslinking of collagen, β_1_ integrin-mediated cell spreading, apoptosis, and proliferation were reduced in HT1080 fibrosarcoma and C2C12 cell lines. This study suggests that EDC crosslinking utilizes the same carboxylic side chain chemistry that is necessary for integrin-mediated cell interactions. This is further substantiated by work confirming that C2C12s highly express β_1_ integrin subunits [103]. Thus, it was hypothesized EDC crosslinking of fibrin microthreads may utilize and subsequently block the functional groups necessary for integrin-mediated cellular attachment.

To uncouple which cellular mechanism, proliferation or migration, is primarily responsible for myoblast outgrowth, Ki67 staining was performed on myoblast-seeded fibrin microthreads from the outgrowth assay. All conditions, regardless of the presence of FGF2 or its incorporation strategy, revealed minimal expression of Ki67^+^ nuclei. Thus, it is likely that the outgrowth observed on fibrin microthreads is driven by myoblast migration, rather than proliferation. Ki67 staining of myoblasts on fibrin microthreads at earlier timepoints during the outgrowth assay would further elucidate the role of cellular proliferation at earlier stages during outgrowth. We hypothesize that minimal Ki67^+^ nuclei were observed because the outgrowth assay was conducted in SFM. There are conflicting reports in the literature regarding whether FGF2 stimulates myoblast proliferation in the absence of serum [97, 104]. Performing this assay in the presence of serum may allow for more robust myoblast outgrowth, as was demonstrated on fibrin microthreads passively adsorbed with hepatocyte growth factor (HGF), another well-known mitogen [63]. In the future, optimization of FGF2 concentration may allow for a more pronounced effect on myoblast proliferation and migration. A dose-dependent effect of FGF2 concentration on C2C12 proliferation has been previously observed [105].

In the future, FGF2-loaded fibrin microthreads could also be investigated for their ability to promote angiogenesis, re-innervation, and fibrosis, which are essential elements of VML regeneration in addition to myogenesis. FGF2 has been shown to stimulate endothelial sprouting, and pericyte and smooth muscle cell migration, allowing FGF2 to stimulate the formation of more mature vessels than other proangiogenic growth factors such as VEGF [32]. Researchers found that heparin-conjugated collagen scaffolds crosslinked with EDC and loaded with FGF2 promoted angiogenesis upon subcutaneous implantation in rats [32, 74, 96]. Fibrin-based scaffolds are also ideal for delivering FGF2 for angiogenesis, as they potentiate FGF2-mediated endothelial cell proliferation [106]. Researchers delivered EDC crosslinked, heparinized, FGF2-loaded fibrin hydrogels to treat murine ischemic hind limb injuries [43]. Injuries treated with FGF2-loaded heparinized fibrin had significantly lower fibrosis and enhanced vascularization compared to controls. Additionally, FGF2 has neurotrophic activity, stimulating NGF synthesis and secretion and promoting neuronal survival and outgrowth [20, 33–36]. Heparinized fibrin hydrogels loaded with FGF2 significantly enhanced neurite length *in vitro* compared to fibrin hydrogels alone [35]. Patel et al. [86] developed aligned poly(*L*-lactide) (PLLA) nanofibers conjugated with heparin to provide sustained release of FGF2, and found these scaffolds stimulated a significantly higher rate of neurite outgrowth compared to unloaded scaffolds. Neurite outgrowth was also enhanced in part due to the aligned, fibrous architecture of this scaffold. This further motivates the use of biopolymer microthread scaffolds with both topographic alignment cues and biomimetic growth factor delivery strategies [107]. In previous studies, we showed that bundles of fibrin microthreads implanted in VML defects in small animal models promote functional skeletal muscle regeneration [18, 108]. In future studies, we will further assess skeletal muscle regenerative using these FGF2-loaded scaffolds in a similar small animal model. If these studies show enhanced functional muscle regeneration, we will use production-scale 3D bioprinting techniques, to increase the size and production rate of these acellular, fibrin microthread-based scaffolds for a large animal porcine VML defect study. Ultimately, these studies are essential to inform the design of pre-clinical studies that are required before translating this therapy into a clinical treatment.

Finally, future work should also investigate FGF2 signaling, as many intracellular signaling pathways are known to be activated by FGF2 including mitogen-activated protein kinases (MAPKs), mechanistic target of rapamycin (mTOR), signal transducer and activator of transcription (STAT), and phospholipase C gamma (PLCγ) [109]. Variations in FGF2 signal transduction pathways explain its pleiotropic nature and better elucidate its role in a variety of regenerative processes including fibrosis, myogenesis, angiogenesis, and innervation. Additionally, this heparin conjugation strategy can also be used to deliver other heparin-binding growth factors, including PDGF, VEGF, bone morphogenetic protein-2 (BMP-2), and HGF, and combinations therein [110].

In the present study, biomimetic fibrin microthread scaffolds were developed to provide physiologically relevant, sustained release of FGF2 by evaluating two incorporation strategies. The first strategy coupled heparin to microthreads through either passive adsorption or covalent carbodiimide coupling, mimicking the presentation of heparan sulfate bound FGF2 in native ECM. Second, we co-incorporated FGF2 by mixing with fibrinogen prior to co-extrusion of microthreads. These strategies sought to leverage the high binding affinity of FGF2 to fibrin, which also protects FGF2 from proteolytic degradation. In these studies, we demonstrated that both FGF2 incorporation strategies achieved sustained release of the bioactive growth factor and stimulated myoblast functions *in vitro*. The results of these studies also suggest that these scaffolds address the limitations of current FGF2 delivery strategies, which primarily use hydrogels and often lack the mechanical strength and alignment cues of microthread scaffolds. We anticipate that these fibrin microthreads which provide mechanical support, topographical alignment cues, and sustained release of FGF2 are a promising scaffold material for treating VML injuries and will be investigated for their ability to promote functional muscle regeneration in future studies.

## Data Availability

The data supporting the conclusions of this manuscript will be made available by the authors, without undue reservation, to any qualified researcher.

## Abbreviations

ANOVA: analysis of variance
BSA: bovine serum albumin
CM: complete medium
co-inc: co-incorporated
dH_2_O: deionized water
ECM: extracellular matrix
EDC: 1-ethyl-3-(3-dimethyl aminopropyl) carbodiimide
ELISA: enzyme-linked immunosorbent assay
FGF2: fibroblast growth factor 2
FTIR: Fourier transform infrared spectroscopy
HEP: heparin sodium salt porcine submucosa
HEPES: *N*-[2-hydroxyethyl]-piperazine-*N*’-[2-ethanesulfonic acid]
Ki67: Kiel 67
NaH_2_PO_4_: sodium phosphate buffer
NHS: *N*-hydroxysuccinimide
PBS: phosphate-buffered saline
PDMS: polydimethylsiloxane
PLGA: poly-lactic-co-glycolic acid
RT: room temperature
SFM: serum-free medium
UNX: uncross linked microthreads
VEGF: vascular endothelial growth factor
VML: volumetric muscle loss
